# Mutational signature of extracranial meningioma metastases and their respective primary tumors

**DOI:** 10.1186/s40478-023-01505-0

**Published:** 2023-01-14

**Authors:** A. Biczok, J. Thorsteinsdottir, P. Karschnia, V. C. Ruf, J. C. Tonn, J. Herms, C. Schichor, M. M. Dorostkar

**Affiliations:** 1 Department of Neurosurgery, Ludwig-Maximilians-University Munich, Marchioninistr. 15, 81377 Munich, Germany; 2 Center for Neuropathology and Prion Research, Ludwig-Maximilians-University Munich, Munich, Germany; 3 German Cancer Research Center (DKFZ), German Cancer Consortium (DKTK), Partner Site Munich, Heidelberg, Germany; 4 Present Address: Department of Pathology, University Clinic of St. Pölten, Karl Landsteiner University of Health Sciences, St. Pölten, Austria

**Keywords:** Meningioma, Metastasis, Molecular alterations, Next-generation sequencing

## Abstract

Extracranial metastases of intracranial meningiomas are rare. Little is known about the mutational pattern of these tumors and their metastatic seeding. Here, we retrospectively explored the molecular alterations of these metastatic lesions and their respective intracranial tumor manifestations.

Histology and genome sequencing were performed in intracranial meningiomas and their extracranial metastatic lesions operated upon between 2002 and 2021. Next-generation DNA/RNA sequencing (NGS) and methylome analysis were performed to determine molecular alterations.

We analyzed the tumors of five patients with clinically suspected metastases of a meningioma using methylome analysis and next generation panel sequencing of the primary tumors as well as the metastatic lesions. Metastases were found in the spinal cord and one in the lung. In four of these patients, molecular analyses confirmed metastatic disease, while the fifth patient was found to harbor two molecularly distinct meningiomas. On pathological assessment, the primary lesions ranged from CNS WHO grades 1 to 3 (integrated molecular-morphologic meningioma classification scores 2 to 6). Of the four true metastatic cases, three out of the four metastasizing tumors harbored alterations in the BAP1 gene, comprising a stop-mutation combined with copy-number loss (WHO grade 1), copy number loss (WHO grade 3) and a frameshift mutation (WHO grade 2). Furthermore, the latter was confirmed to harbor a BAP1 tumor predisposition syndrome. The fourth metastasizing tumor had copy-number losses in NF2 and PTEN. Only one of four showed CDKN2A homozygous deletion; none showed TERT promotor mutation.

Our results molecularly confirm true metastatic disease in four meningioma patients. BAP1 gene alterations were the most frequent. Larger cohorts, most likely from multicenter studies are necessary to evaluate the role of BAP-1 alterations to further understand the metastatic spread in meningiomas. for metastatic spread and might indicate patients at risk for metastatic spread. Further explorations within larger cohorts are necessary to validate these findings which might influence the clinical management in the future.

**Supplementary Information:**

The online version contains supplementary material available at 10.1186/s40478-023-01505-0.

## Introduction

Meningiomas are the most common central nervous system tumors arising from the arachnoid cap cells of the leptomeninges [19]. The vast majority of meningiomas are CNS WHO grade 1 tumors with an excellent long-term outcome after surgical resection. Most patients present with localized disease, where the extent of resection is the strongest clinical prognostic for long-term tumor control and overall survival independent of tumor grade [5, 10, 11]. However, it is rare for meningiomas to metastasize, occurring in less than 1% of all cases and with a higher likelihood in higher grade counterparts [12, 15].

DNA methylation profiling, gene expression signatures, copy-number alterations and histomorphological characteristics are employed to allow a more accurate characterization of these tumors and their potential prognostic implications [1, 6, 7, 18, 24, 25].

Numerous genetic abnormalities have been associated with development of intracranial meningiomas [4, 7, 13, 35]. The most common among these are inactivation of NF2 (moesin-ezrin-radixin like (MERLIN) tumor suppressor) [2], followed by mutations in TRAF7 and KLF4, with the latter two being associated with more benign clinical courses [1]. Within the group of aggressive meningiomas lacking NF2 mutation, in approximately 5% of tumors alterations in chromatin regulators BAP1 and PBRM1 were encountered and correlate with a more aggressive clinical behavior [27, 33]. Additional TERT promoter mutation and homozygous CDKN2A/B loss have similarly been linked to more aggressive meningiomas In contrast, homozygous CDKN2A/B loss, TERT promoter mutations or alterations in BAP1 are associated with more aggressive clinical courses [1, 18, 33].

However, little is known about the genetic profiles of metastasizing meningiomas and their associated clinical behavior. To further stratify and characterize the molecular profile of intracranial meningiomas and their metastatic extracranial lesion we conducted a retrospective study using a next-generation sequencing and methylome analysis.

## Methods

### Study design

In this retrospective analysis we included patients undergoing surgical resection of primary intracranial meningioma and suspected extracranial metastasis at our institution between 2000 and 2021. We screened our database for patients in whom lesions suspicious metastatic meningioma appeared. These Extracranial lesions were identified by whole-body staging using 68 Ga-DOTATATE PET/CT [32]. Tumors were classified according to the most recent WHO classification of tumors of the central nervous system (5th edition, 2021)[17]. Furthermore, the risk score according to the integrated molecular-morphologic meningioma classification was calculated [18]. Demographic, surgical and histologic parameters were retrieved from the medical records.

### Histological evaluation

Neuropathological evaluation was performed on formalin fixed, paraffin embedded tumor tissue using standard H&E stains, immunohistochemical Ki67 staining and staining against trimethylated H3 as well as capillary sequencing of the TERT promoter and copy-number analysis of CDKN2A/B from methylation data. All samples were reclassified in accordance with the most recent WHO classification system [16].

### DNA and RNA isolation

DNA and RNA were extracted from micro-dissected FFPE tissue using an Allprep DNA/RNA Mini Kit (Quiagen, Hilden, Germany) according to the manufacturer’s instructions. Nucleic acid concentrations were determined on a on a Quantus Fluorometer (Promega, Walldorf, Germany) according to the manufacturer’s instructions.

### DNA methylation profiling

Methylation profiling was done using 100–250 ng DNA on an Illumina Infinium MethylationEPIC BeadChip array (Illumina, San Diego, CA, USA), using the plate reader feature of an Illumina NextSeq 550. The protocols were according to the manufacturer’s instructions. Raw methylation data were analyzed using the DNA methylation-based meningioma classifier (version 2.4), which also provides data on copy-number alterations and MGMT promoter methylation [8].

### DNA and RNA sequencing

DNA panel sequencing was performed using the commercially available Illumina Trusight Oncology 500 (TSO500) panel, which is a combined DNA and RNA panel, covering the coding DNA sequence of 522 cancer-associated genes, the TERT promoter, and the RNA sequence of 55 genes to detect fusions and splice variants. Library preparation was performed according to the manufacturer’s instructions using 30–150 ng DNA and 50–85 ng RNA. Sequencing was performed on an Illumina NextSeq 550.

Raw sequencing data were de-multiplexed and aligned to the human genome (GRCh37/hg19) using Illumina software on a dedicated Unix Server, yielding variant calls, DNA amplifications, as well as fusions and splice variants. Variant calls were further evaluated using custom scripts written in Igor Pro (Wavemetrics, Lake Oswego, OR, USA) which retrieved variant effect prediction data from the ENSEMBL server using a REST API. Variants were subsequently filtered based on variant allele frequency (≥ 10%) and minor allele frequency in the population (≤ 1%). Variants annotated as “benign” or “likely benign” in ClinVar were rejected. Variants without a ClinVar entry were filtered both on SIFT and PolyPhen predictions, with variants being rejected which had both SIFT “tolerated” and PolyPhen “benign” as entries. Non-damaging variants present in the dbSNP database which do not have a ClinVar entry were also rejected.

The TERT promoter was additionally analyzed using capillary sequencing, as the coverage on NGS may be low.

## Results

### Patients’ characteristics

**Fig. 1:**
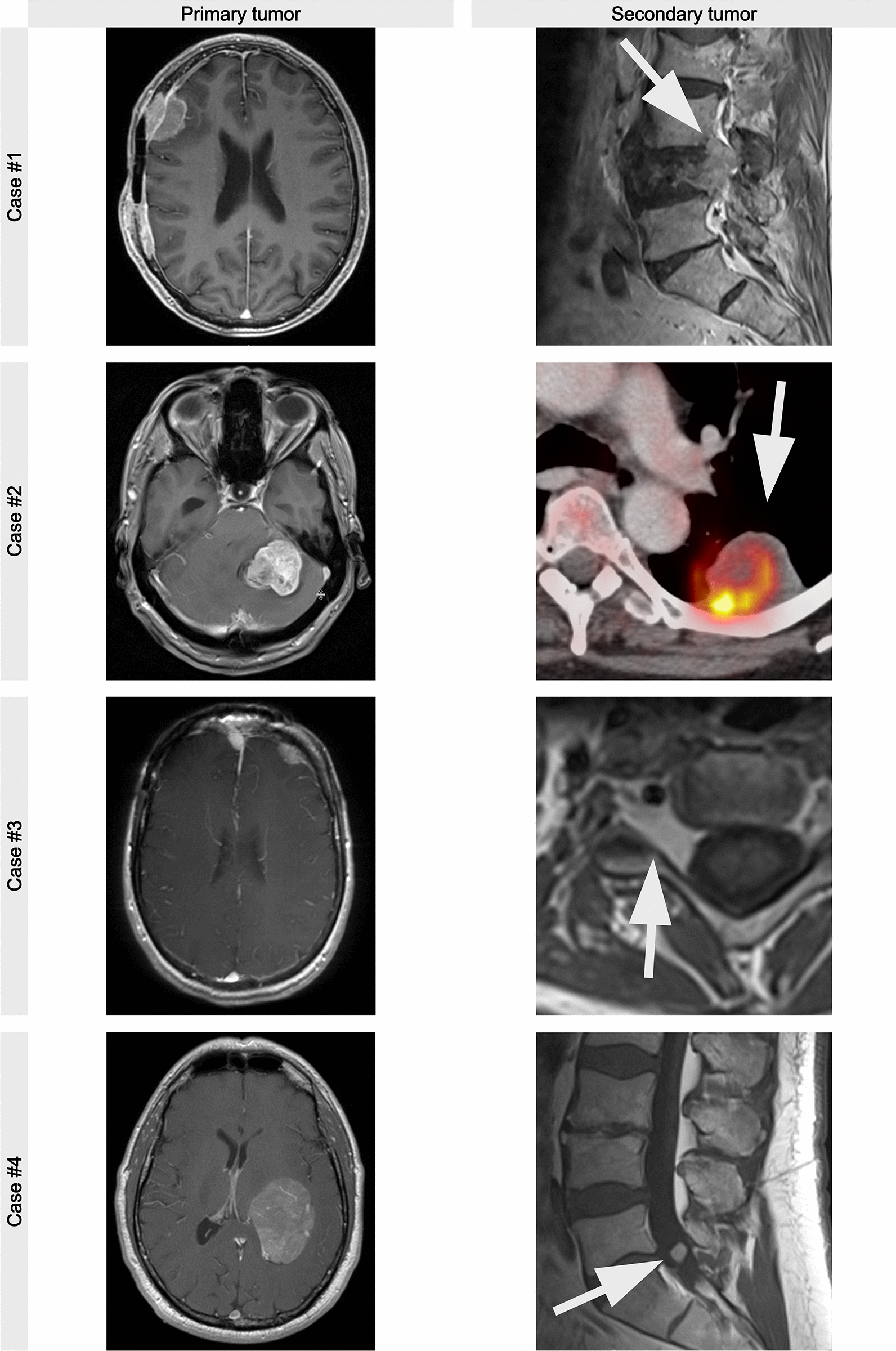
Representative radiographic images of the primary tumor (left column) and respective metastases (right colum)

We performed advanced molecular profiling in a total of 5 patients with suspected extracranial metastatic lesions who had undergone biopsy or resection of both lesions. Histological samples are shown in Fig. 1. The integrated diagnoses of the initial lesions (under consideration of the TERT promoter and CDKN2A/B deletion) were three CNS WHO grade 1 tumors (microcystic, meningothelial and papillary), one CNS WHO grade 2 tumor (atypical) and one anaplastic meningioma, CNS WHO grade 3. The respective integrated molecular-morphologic meningioma classification scores ranged from 2 to 6 (Additional file 3: Table S1) [18].

The median age at diagnosis was 58 years (range: 51–74). Distant metastatic lesions were located in the cervical spinal canal, adjacent to the neuroforamina C3/C4 (case #3) and intradural C4 (case #5) (*n* = 2), lumbar spinal canal adjacent to the lumbar nerves (L5) (*n* = 1), lumbar vertebrae body (L4) (*n* = 1) and intrapulmonary (*n* = 1). Their clinical characteristics are summarized in Table 1.

**Table 1:** Patients characteristics

Patient No.	Age at Diagnosis	Sex	Location	WHO grade	Maas Score
#1 p	54.4	M	Convexity	2	5
#1 m	71.6		Lumbar vertebral body (L4)	2	5
#2 p	59.7	W	Convexity	3	6
#2 m	60.2		Pulmonary	3	6
#3 p	49.7	W	Fronto-basal	1	2
#3 p	51.3		fronto-basal	1	
#3 m	51.4		Cervical spinal canal (neuroforaminal C3/C4)	1	2
#4 p	57.8	M	Intraventricular	3	5
#4 m	58.8		Lumbar spinal canal (intradural adjacent to nerves)	3	3

### Molecular profiles

**Fig. 2:**
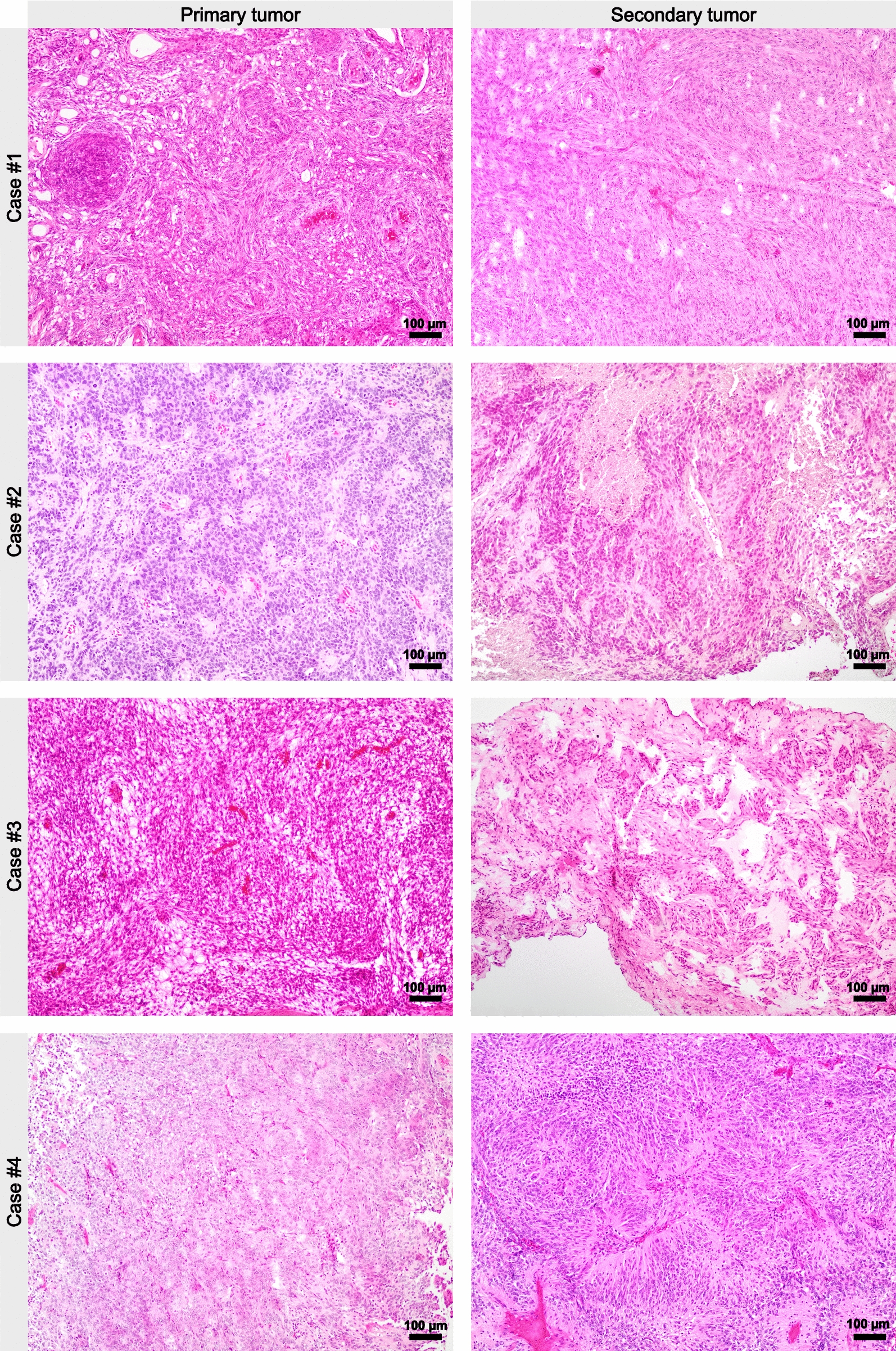
Histomorphology of metastasizing meningiomas. Hematoxylin and eosin-stained slides of the primary tumors (left column) and metastases (right column) are shown. Integrated diagnoses are listed in Additional file 3: Table S1

Molecular analyses revealed identical or overlapping molecular alterations in four of the five patients, thereby confirming metastatic disease. In the tumors of the fifth patient, different molecular alterations were found, which were regarded as evidence for separate tumors (Fig. 2, Additional file 3: Table S1).

Three cases harbored alterations in BAP1 in both the primary and metastatic tumors (case #2, #3 and #4). Among them was one patient who had subsequently been diagnosed with BAP1 tumor predisposition syndrome (patient #4). That patient had a previous history of uveal melanoma and basal cell carcinoma. BAP1 alterations comprised frameshift mutations (case #4), premature stop mutations (case #3) and copy number losses (cases #2 and #3).

**Fig. 3:**
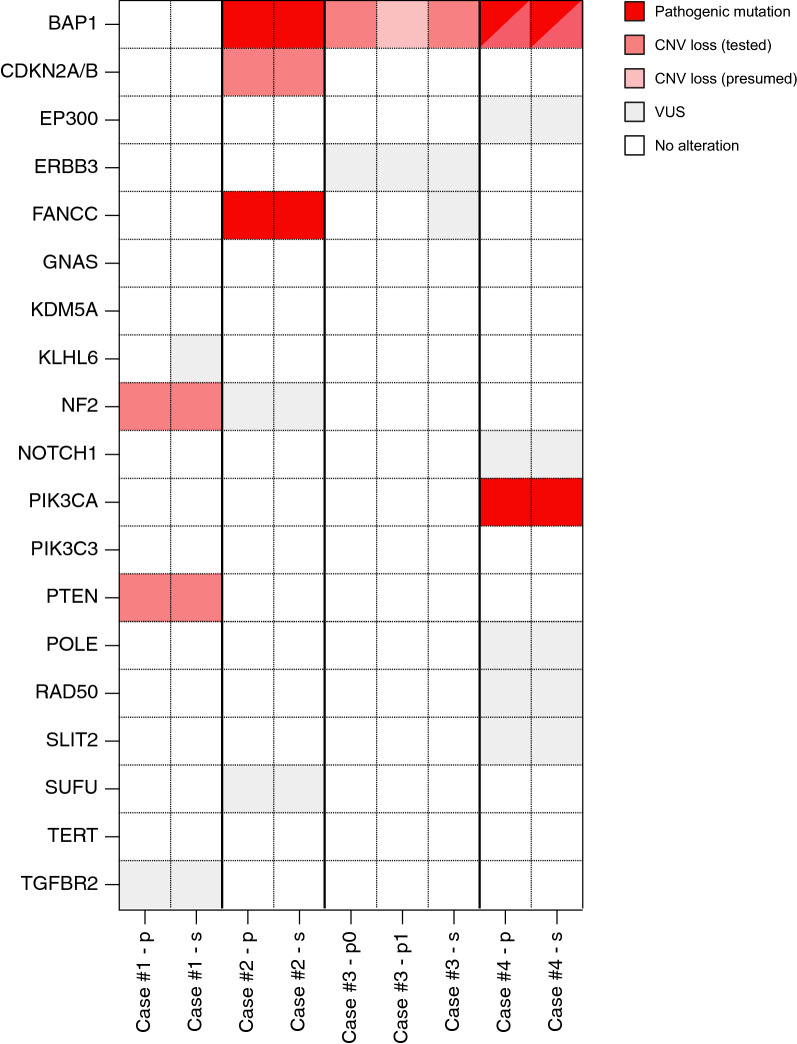
Molecular alterations of metastasizing meningiomas. Oncoplot summarizing molecular findings in primary lesions (labelled p) and secondary lesions/suspected metastases (labelled s). For case #3, a local recurrence at the primary location was also tested. Red color denotes pathogenic alterations, gray variants of uncertain significance (VUS), and tan copy number losses. Variant details are listed in Additional file 3: Table S1

**Fig. 4:**
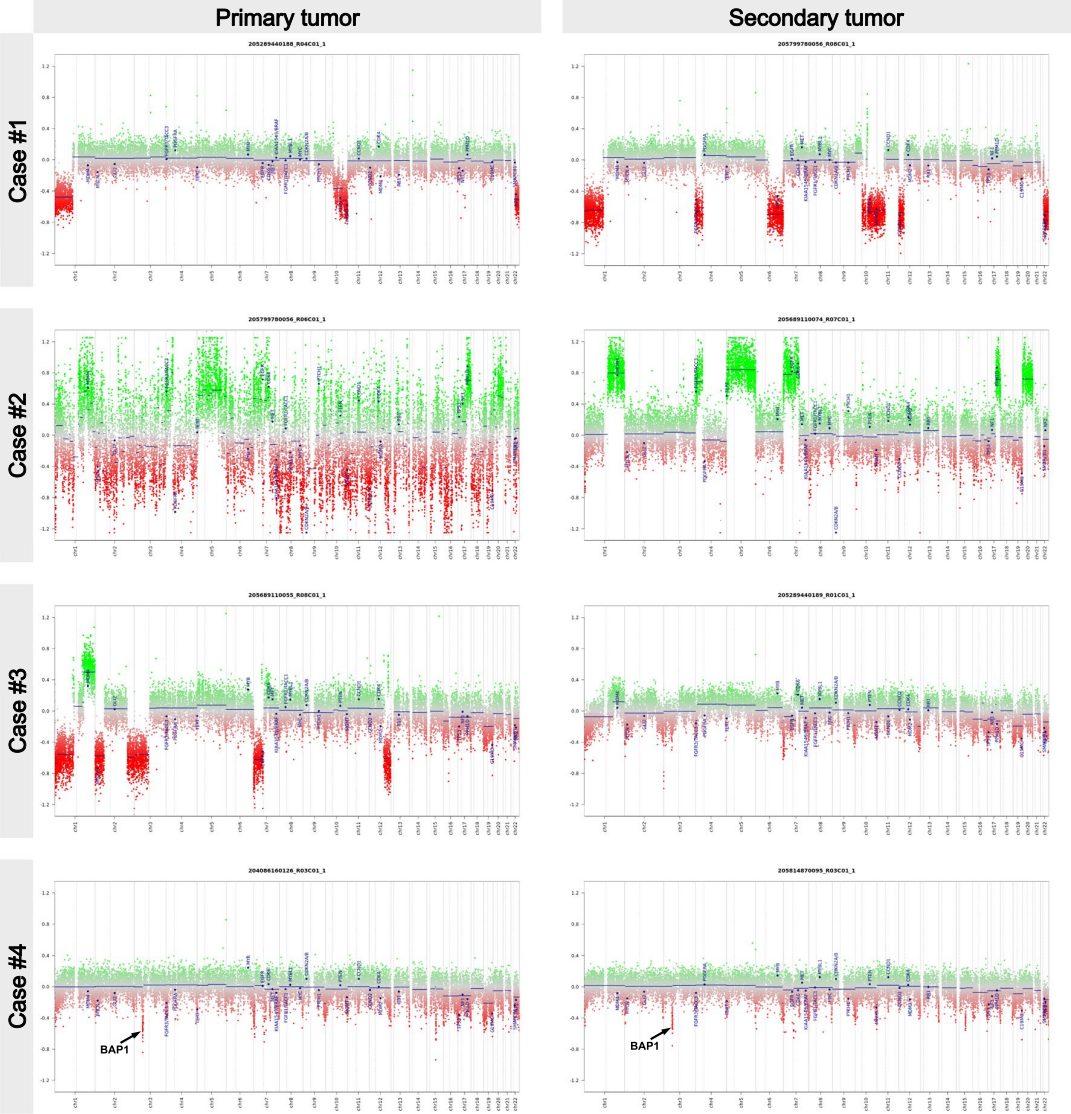
Copy-number plots of metastasizing meningiomas. Primary tumors are shown in the lift column, and metastases in the right. The CNV plot for the primary tumor of case #2 looked identical after renewed DNA isolation and methylome profiling. Note that reduced amplitudes of CNVs in the metastasis of case #3 are likely be due to low tumor cell content in the specimen (cf. Figure 1)

Interestingly, the CNS WHO grades of the three BAP1-altered tumors ranged from 1 to 3, even under consideration of the molecular alterations (Additional file 3: Table S1). Case #2 showed histologically frank malignancy and had a homozygous CDKN2A/B deletion as well as a FANCC frameshift mutation (Figs. 2 and 3). Case #3 had a deletion of the whole chromosome arm 3p, on which BAP1 is located (Fig. 3). Case #4 had a stop mutation in BAP1 as well as a focal copy number loss of BAP1 as well as a PIK3CA mutation (Figs. 2 and 3). The remaining metastasizing case (#1) had copy number losses in NF2 and PTEN and was histologically an atypical meningioma, CNS WHO grade 2 (Fig. 3). Case #5 turned out to harbor different NF2 splice donor variants as well as different bystander mutations in the primary lesion and suspected metastasis and was therefore deemed to have two independent tumors rather than a metastasis. TERT promoter mutations were not found in any of the analyzed samples. Supporting immunohistochemical stains are shown in Additional file 1: Figure S1 and phylogenetic relationships of the studied tumors are shown in Additional file 2: Figure S2 (Fig. 4).

## Discussion

Comparison of the molecular landscape between primary and metastatic lesion have provided insights in numerous entities of cancers. However, a comprehensive knowledge of primary intracranial meningiomas and their distant metastatic lesion could not be established so far.

The goals of this retrospective study were to determine the characteristics of patients with intracranial meningioma and histologically confirmed distant lesions and explore the molecular alterations of these lesions in comparison to their intracranial tumor manifestation.

Recent reports have suggested that individuals inheriting a loss-of-function mutation in breast-cancer (BRCA) 1-associated protein 1 (BAP1) may suffer from several malignancies, mostly uveal melanoma, mesothelioma, clear-cell renal cancer and rhabdoid meningioma. The associated genetic condition is termed BAP1 tumor predisposition syndrome [20, 21, 26, 27, 31]. Recent evidence revealed a crucial role of the BAP1 tumor suppressor gene in a variety of processes including DNA repair mechanisms, transcription, cell death and mitochondrial metabolism [9, 14]. In particular within rhabdoid meningiomas Shankar et al. reported a more aggressive clinical course in BAP1 deficient rhabdoid meningiomas, for both sporadic or familial BAP1 mutations [26]. Furthermore, in rare cases BAP1 mutations was encountered in papillary menigniomas [33]. However, until now there are no clinical studies linking BAP1-mutated meningiomas with distant extracranial metastasis. The majority of patients tested positive for alterations in the tumor suppressor gene BAP1 in both intracranial and distant metastatic lesion. Only one patient (case #4) harbored a germline BAP1 mutation in both intracranial and distant meningioma manifestations, with an additional PIK3CA mutation. Until now PIK3CA was mainly found in transitional/meningothelial meningiomas often located at the skull base, the oncogenic impact has yet to be determined [36]. No other alteration was detected, therefore suggesting that the distant lesion is an identical metastasis. Both tumors were classified as papillary meningiomas (the primary tumor as CNS WHO grade 1 and the metastasis as CNS WHO grade 2). Additionally patient #2 suffered from anaplastic meningioma CNS WHO grade 3 with BAP1 mutation, FANCC mutation and homozygous CDKN2A/B deletion in both manifestations. Interestingly, several studies reported patients harboring a BAP1 mutation in uveal melanoma are at risk for metastasis [28, 29], suggesting a similar risk within meningioma patients.

Molecular studies from other malignancies implicated that tumor consists of multiple clones with unique genomic alterations. These cell-compounds may alter their genomic signature during progression or treatment. Metastatic lesions usually develop from clone-subpopulation of the primary tumor and harbor additional mutations [34].

One of the most common molecular alterations in meningioma are mutations in the NF2 gene, which is located on chromosome 22 and functions as a tumor suppressor gene [22]. In approximately 40–60% of sporadic meningiomas and 80% of high-grade meningiomas, inactivation of NF2 account for tumor development and progression [3, 23, 30]. Two patients harbored an inactivation of NF2. One of these patients has a different splice donor variant in different exons, so this case was deemed not a metastasis but rather a secondary manifestation of a meningioma.

To the best of our knowledge, this is the first study to use next generation sequencing to compare the genetic landscape of intracranial meningiomas with their extracranial lesion in the same patients. Our results suggest that molecular diagnostics allow an accurate discrimination of true metastatic lesions and might indicate an involvement of BAP1 loss-of-function in metastatic spread of meningiomas.

Our study has the inherent limitations of a retrospective analysis. Due to the small sample size and short follow-up period no statement could be made about long-term tumor control. Furthermore, only a small proportion of the tumor tissue was analyzed, so that possible heterogeneity cannot be excluded with certainty. The potential prognostic effect of these identified mutations deserves further evaluation within the framework of larger prospective studies.

In conclusion, the results of this study might provide basis for more accurate prognostic characterizations of meningiomas and their distant (metastatic) lesions. The contribution of alterations within the BAP-1-gene to metastatic spread deserves further investigation within the framework of larger prospective studies.

## Supplementary Information

**Additional file 1: Figure S1**. Immunohistochemical characteristics of metastasizing meningiomas.

**Additional file 2: Figure S2**. Phylogenetic relationships of metastasizing meningiomas in this study.

**Additional file 3**.

## Data Availability

The dataset supporting the conclusions of this article is included within the article as supplementary material. Other data is available from the corresponding author on reasonable request.
