# Supplementary figures and images for: Mutational signature of extracranial meningioma metastases and their respective primary tumors
Source: Acta Neuropathol Commun. 2023 Jan 14;11:12. doi: 10.1186/s40478-023-01505-0 (PMC9840245; doi:10.1186/s40478-023-01505-0)

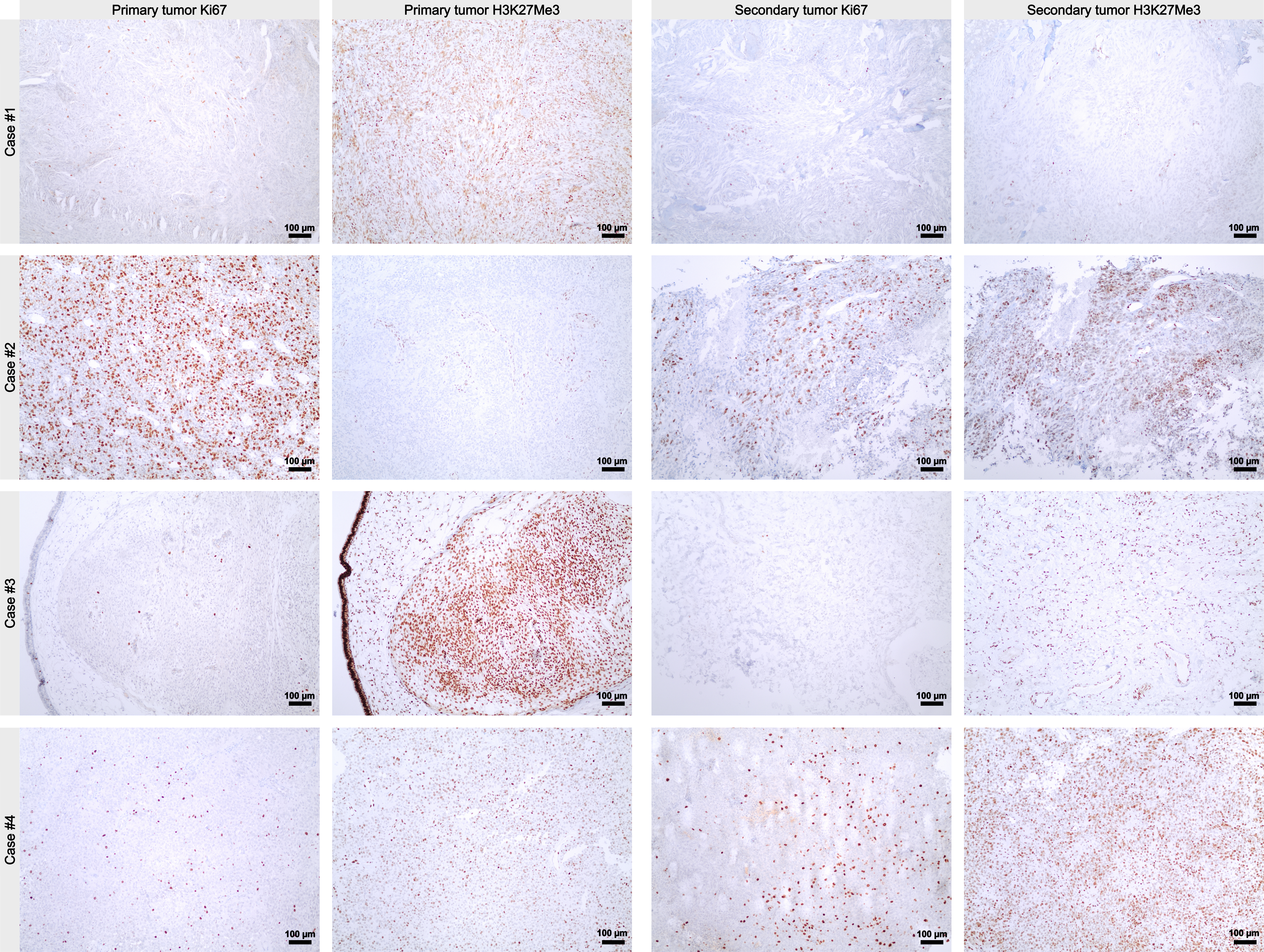

Supplement: Supplementary file 1 — Additional file 1: Figure S1. Immunohistochemical characteristics of metastasizing meningiomas. [file 40478_2023_1505_MOESM1_ESM.pdf]

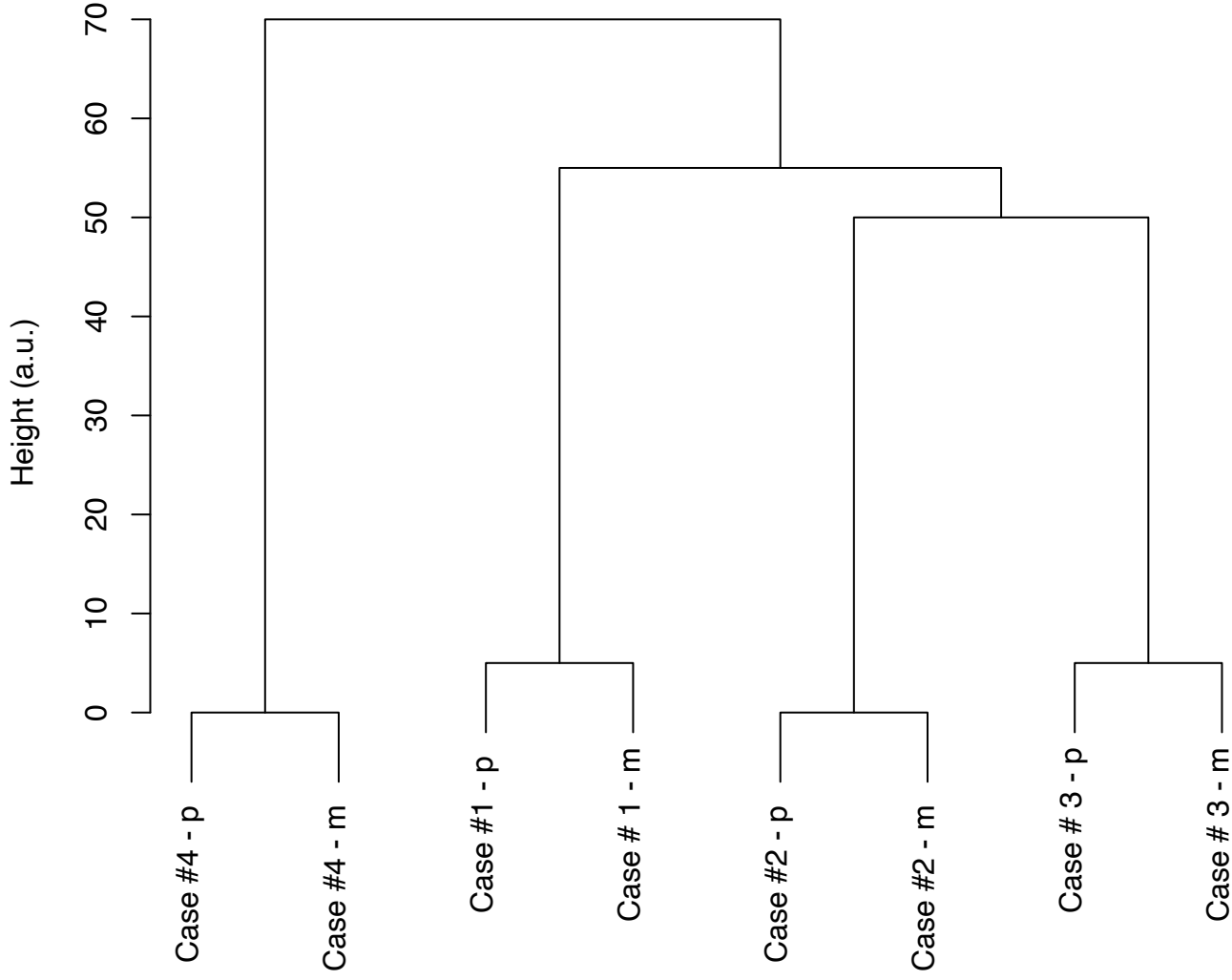

Supplement: Supplementary file 2 — Additional file 2: Figure S2. Phylogenetic relationships of metastasizing meningiomas in this study. [file 40478_2023_1505_MOESM2_ESM.pdf]
